# Comorbidities in pulmonary tuberculosis cases in Puducherry and Tamil Nadu, India: Opportunities for intervention

**DOI:** 10.1371/journal.pone.0183195

**Published:** 2017-08-23

**Authors:** Natasha S. Hochberg, Sonali Sarkar, C. Robert Horsburgh, Selby Knudsen, Jane Pleskunas, Swaroop Sahu, Rachel W. Kubiak, S. Govindarajan, Padmini Salgame, Subitha Lakshminarayanan, Amsaveni Sivaprakasam, Laura F. White, Noyal Maria Joseph, Jerrold J. Ellner, Gautam Roy

**Affiliations:** 1 Section of Infectious Diseases, Department of Medicine, Boston University School of Medicine, Boston, Massachusetts, United States of America; 2 Boston University School of Public Health, Boston, Massachusetts, United States of America; 3 Boston Medical Center, Boston, Massachusetts, United States of America; 4 Department of Preventive and Social Medicine, Jawaharlal Institute of Postgraduate Medical Education and Research, Dhanvantri Nagar, Gorimedu, Puducherry, India; 5 Government Chest Clinic, Puducherry, India; 6 Rutgers University, Newark, New Jersey, United States of America; McGill University, CANADA

## Abstract

**Background:**

We aimed to define characteristics of TB patients in Puducherry and two districts of Tamil Nadu, India and calculate the population attributable fractions (PAF) of TB from malnutrition and alcohol.

**Methods:**

New smear-positive TB cases were enrolled into the Regional Prospective Observational Research for Tuberculosis (RePORT India) cohort. Census and National Family Health Survey data were used for comparisons.

**Results:**

Data were analyzed for 409 participants enrolled between May 2014-June 2016; 307 (75.1%) were male, 60.2% were malnourished (body mass index [BMI] <18.5 kg/m^2^), and 29.1% severely malnourished (BMI <16). “Hazardous” alcohol use (based on AUDIT-C score) was reported by 155/305 (50.8%) of males. Tuberculosis cases were more likely than the Puducherry population to be malnourished (62.6% v 10.2% males and 71.7% v 11.3% of females; both p<0.001), and male cases were more likely to use alcohol than male non-cases (84.4% v 41%; p < .001). The PAF of malnutrition was 57.4% in males and 61.5% in females; the PAF for alcohol use was 73.8% in males and 1.7% in females.

**Conclusions:**

Alcohol use in men and malnutrition are helping drive the TB epidemic in Southern India. Reducing the TB burden in this population will require efforts to mitigate these risk factors.

## Introduction

An estimated 10.6 million cases of tuberculosis (TB) occur annually in the world, and India accounts for 27% of these.[1] The emergence of drug-resistant TB creates a renewed sense of urgency to control the disease. A global expert consultation identified cross-cutting, academic-governmental research partnerships as important, cost-effective platforms for research.[2] Our cohort is part of the Regional Prospective Observational Research for Tuberculosis (RePORT India) Consortium, jointly funded by the Government of India’s Department of Biotechnology, the Indian Council of Medical Research, and the United States National Institutes of Health, and distributed in part by the U.S. Civilian Research & Development Foundation.[3] This Indo-US partnership establishes prospective observational cohorts using clinical, laboratory, and data standards and a linked bio-repository of well-characterized specimens to identify biomarkers of treatment failure and relapse.[3] The use of common data points across RePORT sites in India, South Africa, Brazil and elsewhere will enable comparison and aggregation of data at a supranational level.[3,4]

The WHO End TB strategy has identified the need for “country-specific TB research plans.”[2] In a country as diverse as India, this requires understanding regional differences in the key risk factors for TB. Furthermore, defining characteristics of those who engage in TB care through the public Revised National TB Control Programme (RNTCP) versus private clinics will help programs direct resources. The objectives of this paper are to describe the characteristics of new TB patients seeking care at RNTCP clinics in Puducherry and Tamil Nadu, India; to compare these cases with the overall population and delineate regional characteristics and risk factors; and to define the population attributable fractions (PAF) for key comorbidities. Identification of factors with high population attributable fractions will provide guidance to the local RNTCP on factors that could be addressed to reduce the local burden of TB.

## Materials and methods

### Study setting

The study is an observational household contact (cohort) study conducted by Jawaharlal Institute of Postgraduate Medical Education and Research (JIPMER) in collaboration with Boston University Medical Campus and Rutgers University. In Puducherry (population ~9.5million), there is one TB Unit (TU), and enrollment began in May 2014. Of the 32 districts of Tamil Nadu, Cuddalore (population 2.6 million) and Villapuram (population 3.5 million) are adjacent to Puducherry; two TUs were selected in each district (population served is approximately 500,000 per TU). Enrollment began in Cuddalore in August 2014 and Villapuram in November 2015.

### Study design

Patients were recruited from RNTCP district microscopy centers and primary healthcare centers. Written informed consent was obtained from all individuals meeting study inclusion criteria who consented to participate. Inclusion criteria include: 1. Newly diagnosed smear-positive (at least 1+ acid fast bacilli [AFB]), culture-confirmed pulmonary TB. 2. Having taken < three doses of anti-TB medication. 3. No history of TB or TB treatment. 4. Plan to stay in study area for the study duration. 5. >5 years of age. Exclusion criteria include: 1. Unwilling to be tested for HIV. 2. Diagnosis of or contact with someone with known multi-drug resistant-TB. 3. Choose not to initiate TB treatment; or 4. Too sick to enroll, defined as a Karnofsky score ≤ 10 (moribund).[5]

### Study procedures

Upon enrollment, study personnel administered a questionnaire to assess demographic and clinical characteristics including age, gender, marital status, religion, caste, education level, other socioeconomic characteristics, TB symptoms and duration, comorbidities, and Karnofsy score. [5] Participants were asked about alcohol use using the Alcohol Use Disorders Identification Test (AUDIT)-C questionnaire (a modified version of AUDIT),[6] and smoking, including the product (e.g., bidis, store bought cigarettes), frequency and duration of use. Baseline anthropometric measurements included body mass index (BMI) and mid upper arm circumference. One follow-up visit was performed between weeks 8–12 and another at treatment completion. Questions were translated into Tamil and administered by Tamil-speaking interviewers. A random sputum sample was collected at enrollment for concentrated AFB smear and Löwenstein–Jensen and mycobacterial growth indicator tube (MGIT) cultures.

### Measurements and definitions

Diabetes was defined as random blood sugar >200mg/dL or self-report; severe malnutrition was defined as BMI <16 kg/m^2^ and malnutrition as BMI 16–18.5 kg/m^2^. Anemia was defined as hemoglobin < 13 g/dl for men and < 12 g/dl for women.[7] “Hazardous” alcohol use was defined as per AUDIT-C.[8] Scheduled caste is the lowest caste, and members are awarded special government benefits; “other backwards caste” is above scheduled caste but below all others.[9]

### Data sources

Population data came from the Indian 2011 census (for demographics and assets)[10] and the 2015 National Family Health Survey (NFHS) (for health, BMI and alcohol data).[7] The NFHS reported any current alcohol use (not hazardous use). This study protocol was reviewed and approved by the Boston University Medical Campus and Rutgers Institutional Review Boards. The study was also approved by the JIPMER Ethics Committee and Scientific Advisory Committee. Consent was obtained from participants; for minors, assent was obtained as well as parental consent.

### Data management and statistical analysis

Paper questionnaires were scanned, sent to Boston using Verity TeleForm Information Capture System software V10.8 (Sunnyvale, CA, USA), and read into a Microsoft Access (Seattle, WA, USA) database. Errors were reviewed and corrected by the on-site team. Data analyses were performed using SAS V9.4 (Cary, NC, USA). Dichotomous variables were compared using the chi square test and continuous variables using the two-sample two-sided t-test. Comparisons to the census and NFHS data were made and p-values calculated using chi-square tests for differences in proportions between the two samples. The PAFs for malnutrition and alcohol use in Puducherry alone were calculated for those aged 15–49 years using the equation PAF = Ppop*(RR-1)/[(Ppop*(RR-1)+1] where Ppop is the prevalence of the exposure in the population (based on NFHS data) and RR is the relative risk of TB (using study data for Puducherry and the 2011 census data for Puducherry)[11]. Because the NFHS included only persons 15–49 years old, the comparison analysis was performed using study data for those aged 15–49 years. The PAFs do not control for potential confounders or effect modifiers.

## Results

### Demographics

Of the 549 smear-positive TB patients evaluated in RNTCP clinics between May 2014-June 2016 and screened for study enrollment, 409 (74.5%) consented to participate. Of the 140 not enrolled, 135 (96.4%) were ≥18 years of age and 105 (75%) male. Of those enrolled, 307/409 (75.1%) were male, the mean age was 45 years (range 14–81), and 283 (69.9%) were married (Table 1). Almost all (98.0%) participants were from a scheduled or other backwards caste. Index cases had a median of 7 years of education (range 0–18) and their mothers a median of 0 years (range 0–15). Over half (54.3%) of cases first sought care at a private clinic. Households had a median of 4 people (range 1–13). Household characteristics were available for 185 index cases for whom household contacts were also enrolled. Monthly household income was <5000 rupees (approximately $79) for 52.6%.

**Table 1:** Characteristics of TB patients in RePORT cohort, Puducherry and Tamil Nadu, (2014–16).

	Male, n (%) (n = 307)	Female, n (%) (n = 102)	Total, n (%) (n = 409)
***Demographic Characteristics***			
Male gender	307 (100)	0 (0)	307 (75.1)
Age (mean, range)	47 (17–81)	34 (14–75)	45 (14–81)
Marital status			
Married/living together	243/305 (79.7)	40/100 (40.0)	283/405 (69.9)
Never married	33/305 (10.8)	31/100 (31.0)	64/405 (15.8)
Widowed	18/305 (5.9)	26/100 (26.0)	44/405 (10.9)
Separated/divorced	11/305 (3.6)	3/100 (3.0)	14/405 (3.5)
Caste/Tribe^a^			
Other backwards caste	222/305 (72.8)	74/100 (74.0)	296/405 (73.1)
Scheduled Caste	77/305 (25.2)	24/100 (24.0)	101/405 (24.9)
None of them	3/305 (1.0)	2/100 (2.0)	5/405 (1.23)
Don’t Know	3/305 (1.0)	0 (0)	3/405 (0.74)
Employment/occupation			
Employed	282/304 (92.8)	41/100 (41.0)	323/404 (80.0)
Unemployed	12/304 (3.95)	37/100 (37.0)	49/404 (12.1)
Student	7/304 (2.3)	22/100 (22.0)	29/404 (7.2)
Other	3/304 (0.99)	0 (0)	3/404 (0.74)
Education, years; median (range)	7 (0–18)	9 (0–17)	7 (0–18)
Maternal education, years; median (range)	0 (0–12)	0 (0–15)	0 (0–15)
No. people in household; median (range)	4 (1–9)	4 (1–13)	4 (1–13)
Monthly household income			
<Rs 3000 ($48)	50/305 (16.4)	11/100 (11.0)	61/405 (15.1)
Rs 3000–5000 ($48–79)	108/305 (35.4)	44/100 (44.0)	152/405 (37.5)
Rs 5001–10000 ($79–158)	108/305 (35.4)	29/100 (29.0)	137/405 (33.8)
> Rs 10000 (>$158)	34/305 (11.1)	11/100 (11.0)	45/405 (11.1)
Refused to Answer	1/305 (0.3)	3/100 (3.0)	4/405 (1.0)
Don’t Know	4/305 (1.3)	2/100 (2.0)	6/405 (1.5)
***Comorbidities***			
Smoking status			
Current smoker	60/305 (19.7)	0 (0)	60/405 (14.8)
Former smoker	136/305 (44.6)	1/100 (1.0)	137/405 (33.8)
Alcohol			
Any use	246/305 (80.7)	2/100 (2.0)	248/405 (61.2)
Hazardous use^b^	155/305 (50.8)	2/100 (2.0)	157/405 (38.8)
BMI, kg/m^2^			
<16	91/305 (29.8)	27/100 (27.0)	118/405 (29.1)
16–18.5	94/305 (30.8)	32/100 (32.0)	126/405 (31.1)
18.6–24.9	109/305 (35.7)	32/100 (32.0)	141/405 (34.8)
25–29.9	10/305 (3.3)	8/100 (8.0)	18/405 (4.4)
>30	1/305 (0.3)	1/100 (1.0)	2/405 (0.5)
Diabetes mellitus			
Self-report	90/291 (30.9)	28/96 (29.2)	118/387 (30.5)
Random blood sugar >200 mg/dL	86/305 (28.2)	27/100 (27.0)	113/405 (27.9)
Self-report or random blood sugar	111/305 (36.4)	33/100 (33.0)	144/409 (35.2)
HIV-infected (n = 383)	0 (0)	1 (1.1)	1 (0.3)

^a^ Scheduled castes are the lowest caste and receive government benefits. Other backwards caste is a collective term to classify castes that are socially and educationally disadvantaged.

^b^ Hazardous alcohol use is defined based on AUDIT-C score.

### Comorbidities

Among males, 60 (19.7%) were current smokers and 136 (44.6%) former smokers; only one female was a former smoker and none were current smokers. Any alcohol use was reported by 246 (80.7%) males and two (2.0%) females and hazardous alcohol use by 155 (50.8%) and two (2.0%), respectively. Among TB cases, 60.2% were malnourished including 29.1% who were severely malnourished. In total, 144 (35.2%) had diabetes as determined by self-report (n = 118 [30.5%]) and/or random blood sugar >200 mg/dL (n = 113 [27.9%]).

### Comparison with population and population attributable fractions

Comparing the TB cases in the RePORT cohort and the Puducherry population, the former had a greater proportion of males (75.1% vs 49.1%, p<0.001) and tended to be older (Table 2). Data on household income were not available from the Census or NFHS, so we compared other measures of socioeconomic status. A larger proportion in RePORT were scheduled caste than in the population (26.4% vs. 15.7%; p = 0.06), more had cement floors (72.4% vs 56.5%, p = 0.02), and fewer had tile floors (16.2 vs. 29; p = 0.03) or a private water source (45.7% vs. 80.5%; p<0.001). The TB cases were more likely to be malnourished than the Puducherry general population (71.7% vs. 11.3% of females; p<0.001, and 62.6% vs. 10.2% of males; p < .001; Table 3). Male TB cases were also more likely to use alcohol (84.4% of study population vs. 41% in Puducherry; p<0.001). The PAF of malnutrition was 57.4% in males and 61.5% in females; the PAF for alcohol use was 73.8% in males and 1.7% in females.

**Table 2:** Comparison of TB case characteristics, household characteristics and Puducherry population based on 2011 census data.

	Study cohort (n = 409)	Puducherry population (%)	p-value	OR (95% CI)
Male, %	75.1	49.1	<0.001	3.26 (1.8–5.9)
Age categories, years %				
< 20	16.1	33.9	<0.001	0.37 (0.2–0.7)
20–29	11.5	18.5	0.17	0.57 (.3–1.3)
30–39	13.7	17.3	0.48	0.75 (.4–1.6)
40–49	22.9	13.1	0.07	1.9 (0.9–4.2)
50–59	19.9	8.6	0.02	2.6 (1.1–6.2)
60–69	13.0	5.3	0.06	2.7 (0.9–7.6)
70–79	2.7	2.3	0.86	1.2 (0.2–7.0)
80+	0.22	1.0	0.48	0.2 (<0.1–24.3)
Scheduled caste, %	26.4	15.7	0.06	1.9 (1.0–3.9)
Marital Status, %				
Never married	16.4	27.6	<0.001	0.5 (0.3–1.0)
Married	69.4	50.7	<0.001	2.2 (1.2–3.9)
Other	14.3	7.5	0.12	2.1 (0.8–5.2)
Mean number of household members	3.5	4.1	<0.001	
Assets				
Bicycle	70.4	52.2	0.01	2.2 (1.2–3.9)
Phone^a^	98.9	82.5	<0.001	19.1 (2.7–133.9)
Radio	19.4	25.0	0.34	0.7 (0.4–1.4)
TV	98.4	84.0	<0.001	11.7 (2.2–61.1)
Car	0	5.7	0.02	NA
Ownership of home	61.3	64.3	0.66	0.9 (0.5–1.6)
Roof material				
Thatch/Grass	20.5	17.4	0.58	1.2 (0.6–2.5)
Concrete	53.5	64.0	0.13	0.7 (0.4–1.1)
Asbestos sheets	16.7	7.9	0.06	2.3 (1.0–5.7)
Other	9.2	10.7	0.72	0.8 (0.3–2.1)
Floor material				
Cement	72.4	56.5	0.02	2.0 (1.1–3.6)
Tile	16.2	29.0	0.03	0.5 (0.2–0.9)
Mud/Earth/Sand	7.0	9.0	0.47	0.8 (.3–2.1)
Other	4.3	5.5	0.69	0.8 (0.2–2.8)
Water source for home				
Private	45.7	80.5	<0.001	0.2 (0.1–0.4)
Near premises	45.2	18.7	<0.001	3.6 (1.9–6.8)
Other	9.1	0.8	0.007	12.4 (1.2–124.2)

^a^ Mobile phone or land-line telephone

**Table 3:** Comparison of TB case characteristics and the 2015 national family health survey for Puducherry for persons ages 15–49 years^a^.

	Study cohort (%)	Puducherry population (%)	p-value	OR (95% CI)
BMI <18.5, %				
Women	71.7	11.3	<0.001	19.9 (9.3–42.4)
Men	62.6	10.2	<0.001	14.7 (6.9–31.6)
BMI > 25, %				
Women	6.7	36.7	<0.001	0.12 (0.1–0.3)
Men	3.9	37.1	<0.001	0.07 (0.02–0.2)
Current Smokers, %				
Women	0	1.1	0.62	NA
Men	18.9	14.4	0.39	1.4 (0.7–2.9)
Alcohol Use, % ^b^				
Women	1.7	0.6	0.23	2.9 (0.2–55.1)
Men	84.4	41.0	<0.001	7.8 (3.9–15.2)

^a^National Family Health Survey (NFHS) Comparison Data only includes those that are aged 15–49; study population includes only those in the same age category

^b^NFHS reports any alcohol use; comparable data are reported from the study cohort

### Clinical and microbiologic data

Individuals were symptomatic for a median of 4 weeks (range 1–12) before starting treatment; the symptom of longest duration was weight loss. All but two had cough (406; 99.5%), and median cough duration was 4 weeks (range 1–7). Overall, 374 (92.1%) reported weight loss.

## Discussion

This study uses the JIPMER RePORT cohort to define the characteristics of new smear-positive pulmonary TB patients in Puducherry and Tamil Nadu, India who access RNTCP clinics. These TB patients tend to be male, of poor socioeconomic status (based on household income, home construction and access to water) and malnourished; many of the men are hazardous alcohol users. In this region, malnutrition and alcohol use, both potentially reversible risk factors, are key drivers of TB disease. Defining the characteristics of TB patients in Puducherry and Tamil Nadu allows for targeted public health programs which may vary regionally in a country as diverse as India.

Among our male patients age 15–49 years, the PAF of alcoholism is 73.8%. In India as a whole, the PAF of TB due to alcohol “misuse” has previously been calculated to be 5–6.9%,[12,13] but our data suggest this association is far stronger in Puducherry and Tamil Nadu. In this region, it is possible that up to three-quarters of male TB cases could be eliminated if the impact of alcohol were mitigated. The striking differences in alcohol use (and hazardous alcohol use) may also explain some of the sex imbalances in TB cases in India and in our study population (where the PAF for alcohol in women was only 1.7%).[14] Alcohol treatment programs are few in this area and more may be needed;[15] by reducing alcohol use, TB cases would be fewer.[12,16] Furthermore, alcohol use results in poor TB treatment adherence and worse TB outcomes, including death;[17,18] closer integration between TB control programs and alcohol treatment programs may improve outcomes.[19–21] Currently RTNCP recommends practitioners elicit a history of alcohol use and advise patients to abstain from alcohol.[21] Use of the AUDIT-C scale in RNTCP programs could improve detection of hazardous alcohol use and identify patients that need directed services.[16] Notably, alcohol use among TB cases varies widely in India with rates of 29% in Chennai[20] and 20% in Karnataka.[22] Differences in alcohol use by location may reflect the cost, as the price and taxation rate (e.g., value added tax [VAT]) vary by state (S. Sarkar, personal communication). The integration of alcohol treatment into TB programs may therefore be less critical in some areas of India, but it certainly could have value for our site.

Among our patients, the PAF of malnutrition is 57.4% in males and 61.5% in females age 15–49 years. The high PAF of TB due to malnutrition is of particular importance, because India has a high burden of malnutrition (greater than one-third of those age 15–49 years).[23] By comparison, in 22 high TB-burden countries, the PAF of malnutrition has been reported to be only 26.9%.[12] It is plausible that some of the malnutrition is secondary to TB itself (particularly given the high rates of weight loss reported by participants), but malnutrition is also a clear risk factor for TB. Many studies have found increased TB incidence from malnutrition, including a 12.4-fold increased hazard of developing TB for those with BMI <18.5 kg/m^2^ and a log-linear relationship between TB incidence and BMI.[24–26] The known impact of malnutrition on TB disease severity, treatment outcomes, relapse, and mortality suggest that TB programs, particularly in countries such as India, should address nutrition.[24–26] This effort will require enhanced collaboration between government sectors, as “social protection, poverty alleviation, and actions on other determinants of TB” are critical components of the EndTB strategy.[27]

Although we did not have comparison data to calculate the regional PAF for diabetes mellitus and TB, the high prevalence in this cohort is of serious concern given the increased risk of TB and rising prevalence in India.[28] Diabetes is found in more than 65.1 million people in India (second only to China in absolute numbers),[29] and approximately one-third of TB cases in India;[30] country-wide, 9.1% of TB cases are attributable to diabetes.[12] Diabetes not only increases the risk of TB infection and progression to active TB but also contributes to treatment failure, relapse, death.[31,32] Our data also suggest that almost 1/5 diabetics are not aware of their diagnosis; increased testing may be warranted.

The fact that one-third of our cohort were former smokers and 14.8% currently smoked will have implications for TB outcomes. Smoking exacerbates the long-term damage caused by TB including fibrosis, bronchiectasis, and chronic obstructive pulmonary disease.[33,34] Furthermore, it is concerning that 14.4% of males in the greater Puducherry population smoke, because smokers have an increased odds of *Mycobacterium tuberculosis* infection and active TB disease.[35]

There are several strengths and limitations to this study. Study strengths include the high participation rate and the fact that the study population reflects the majority of new smear-positive pulmonary TB cases seeking care at RNTCP clinics in the catchment area. Furthermore, the use of validated questionnaires (e.g. AUDIT-C) to assess demographics and comorbidities will allow for valuable comparisons with other cohorts in India and elsewhere. The interpretation of PAFs is limited by the fact that the study cohort appears to be a poorer subset of the Puducherry and Tamil Nadu populations, and socioeconomic status may confound the association between TB and malnutrition and alcohol. Furthermore, we used comparator census and NFHS data from Puducherry alone and not Tamil Nadu to reflect the distribution of cases (78.5% are from Puducherry). The study populations and cases in these neighboring areas are very similar when comparing census data on literacy and household condition, among other factors.[7,10] Moreover, available census and NFHS data are from 2011 and 2015, respectively. It is possible that population characteristics changed somewhat between 2011 and 2015; however, we doubt such changes were substantial enough to have altered our findings.

## Conclusion

This analysis provides insight into the characteristics of new smear-positive TB patients accessing care through RNTCP in Puducherry and Tamil Nadu and the drivers of TB in this region. As attention is increasingly paid to the division between public and private TB care in India, our results are most relevant to those receiving care from RNTCP. In this population, recognizing the important role of alcohol use, malnutrition, diabetes, and other factors will enable public health programs to target region-specific high-risk conditions in the fight against TB.

## Supporting information

S1 File De-identified dataset of variables included in the analysis. (SAS7BDAT)

## References

[1] World Health Organization (WHO). Global Tuberculosis Report 2016. Geneva, Switzerland; 2016.

[2] Lienhardt C, Lonnroth K, Menzies D, Balasegaram M, Chakaya J, Cobelens F, et al Translational Research for Tuberculosis Elimination: Priorities, Challenges, and Actions. PLoS Med. 2016;13: e1001965 doi: 10.1371/journal.pmed.1001965 26933883 10.1371/journal.pmed.1001965 PMC4775029

[3] Hamilton CD, Swaminathan S, Christopher DJ, Ellner J, Gupta A, Sterling TR, et al RePORT International: Advancing Tuberculosis Biomarker Research Through Global Collaboration. Clin Infect Dis. 2015;61: 155–159. doi: 10.1093/cid/civ227 26409277 10.1093/cid/civ611 PMC4583572

[4] Geadas C, Stoszek SK, Sherman D, Andrade BB, Srinivasan S, Hamilton CD, et al Advances in basic and translational tuberculosis research. Tuberculosis. Elsevier; 2017;102: 55–67. doi: 10.1016/j.tube.2016.11.006 28061953 10.1016/j.tube.2016.11.006 PMC9067314

[5] Karnofsky Performance Status. [Internet]. Available: https://www.cancer.gov/publications/dictionaries/cancer-terms?cdrid=44156

[6] Babor T, Higgins-Biddle JC, Saunders JB, Monteiro MG. The Alcohol Use Disorders Identification Test. World Heal Organ Dep Ment Heal Subst Depend. 2001;

[7] National Family Health Survey, India, 2015–2016. State Fact Sheet Puducherry [Internet]. Available: http://rchiips.org/NFHS/pdf/NFHS4/PY%7B_%7DFactSheet.pdf

[8] Frank D, DeBenedetti AF, Volk RJ, Williams EC, Kivlahan DR, Bradley KA. Effectiveness of the AUDIT-C as a Screening Test for Alcohol Misuse in Three Race/Ethnic Groups. J Gen Intern Med. New York: Springer-Verlag; 2008;23: 781–787. doi: 10.1007/s11606-008-0594-0 18421511 10.1007/s11606-008-0594-0 PMC2517893

[9] Ministry of Social Justice and Empowerment. Scheduled Caste Welfare. [Internet]. [cited 10 Nov 2016]. Available: http://socialjustice.nic.in/UserView/index?mid=19536

[10] 2011 Census Data [Internet]. [cited 15 Oct 2016]. Available: http://censusindia.gov.in/

[11] Rothman KJ, Greenland S. Modern Epidemiology, 2nd Edition Philedelphia, PA: Lippincott Williams {&} Wilkins Publishers; 1998.

[12] Lönnroth K, Castro KG, Chakaya JM, Chauhan LS, Floyd K, Glaziou P, et al Tuberculosis control and elimination 2010–50: cure, care, and social development. Lancet. Elsevier; 2017;375: 1814–1829. doi: 10.1016/S0140-6736(10)60483-7 10.1016/S0140-6736(10)60483-7 20488524

[13] Kolappan C. Selected biological and behavioural risk factors associated with pulmonary tuberculosis. Int Lung Dis. 2007;11: 999–1003. 17705978

[14] Horton KC, MacPherson P, Houben RMGJ, White RG, Corbett EL. Sex Differences in Tuberculosis Burden and Notifications in Low- and Middle-Income Countries: A Systematic Review and Meta-analysis. PLOS Med. Public Library of Science; 2016;13: e1002119 Available: doi: 10.1371/journal.pmed.1002119 27598345 10.1371/journal.pmed.1002119 PMC5012571

[15] Sujiv A, Chinnakali P, Balajee K, Lakshminarayanan S, Kumar SG, Roy G. Alcohol use and alcohol use disorder among male outpatients in a primary care setting in rural Puducherry. Ind Psychiatry J. India: Medknow Publications {&} Media Pvt Ltd; 2015;24: 135–139. doi: 10.4103/0972-6748.181711 27212816 10.4103/0972-6748.181711 PMC4866339

[16] Thomas B, Suhadev M, Mani J, Ganapathy BG, Armugam A, Faizunnisha F, et al Feasibility of an alcohol intervention programme for TB patients with alcohol use disorder (AUD)—a qualitative study from PLo;:e27752 doi: 10.1371/journal.pone.0027752 Epub Nov 21. 2011;6. 10.1371/journal.pone.0027752 PMC3221662 22132134

[17] Lonnroth K, Williams BG, Stadlin S, Jaramillo E, Dye C. Alcohol use as a risk factor for tuberculosis—a systematic review. BMC Public Health. 2008;8: 289 doi: 10.1186/1471-2458-8-289 18702821 10.1186/1471-2458-8-289 PMC2533327

[18] Santha T, Garg R, Frieden TR, Chandrasekaran V, Subramani R, Gopi PG, et al Risk factors associated with default, failure and death among tuberculosis patients treated in a DOTS programme in Tiruvallur District, South India, 2000. Int J Tuberc Lung Dis. France; 2002;6: 780–788. 12234133

[19] Mathew TA, Yanov SA, Mazitov R, Mishustin SP, Strelis AK, Yanova G V, et al Integration of alcohol use disorders identification and management in the tuberculosis programme in Tomsk Oblast, Russia. Eur J Public Health. 2009;19: 16–18. doi: 10.1093/eurpub/ckn093 19112073 10.1093/eurpub/ckn093 PMC2720714

[20] Suhadev M, Thomas BE, M RS, P M, V C, Charles N, et al Alcohol Use Disorders (AUD) among Tuberculosis Patients: A Study from Chennai, South India. PLoS One. Public Library of Science; 2011;6: e19485 doi: 10.1371/journal.pone.0019485 21611189 10.1371/journal.pone.0019485 PMC3096635

[21] Revised National Tuberculosis Control Programme: Training Course for Program Manager. [Internet]. Available: http://www.tbcindia.nic.in/index1.php?lang=1%7B&%7Dlevel=3%7B&%7Dsublinkid=4262%7B&%7Dlid=2906

[22] Thapa P, Kamath R, Shetty BK, Monteiro A, Sekaran VC. Prevalence and Associated Factors of Alcoholism among Tuberculosis Patients in Udupi Taluk, Karnataka, India: A Cross Sectional Study. J Nepal Health Res Counc. Nepal; 2014;12: 177–181. 26032055

[23] Bhargava A, Benedetti A, Oxlade O, Pai M, Menzies D. Undemutrition and the incidence of tuberculosis in India: National and subnational estimates of the population-attributable fraction related to undemutrition. Natl Med J INDIA. 2014;27: 128–133. 25668081

[24] Cegielski JP, Arab L, Cornoni-Huntley J. Nutritional Risk Factors for Tuberculosis Among Adults in the United States, 19711992. Am J Epidemiol. United States; 2012;176: 409–422. doi: 10.1093/aje/kws007 22791739 10.1093/aje/kws007 PMC5788452

[25] Lönnroth K, Williams BG, Cegielski P, Dye C, Lonnroth K, Williams BG, et al A consistent log-linear relationship between tuberculosis incidence and body mass index. Int J Epidemiol. World Health Organization, Stop TB Department, Geneva, Switzerland. lonnrothk@who.int; 2010;39: 149–155. doi: 10.1093/ije/dyp308 19820104 10.1093/ije/dyp308

[26] Zachariah R, Spielmann MP, Harries AD, Salaniponi FML. Moderate to severe malnutrition in patients with tuberculosis is a risk factor associated with early death. Trans R Soc Trop Med Hyg. England; 2002;96: 291–294. 12174782 10.1016/s0035-9203(02)90103-3

[27] Padmapriyadarsini C, Shobana M, Lakshmi M, Beena T, Swaminathan S. Undernutrition & tuberculosis in India: Situation analysis & the way forward. Indian J Med Res. India: Medknow Publications & Media Pvt Ltd; 2016;144: 11–20. doi: 10.4103/0971-5916.193278 27834321 10.4103/0971-5916.193278 PMC5116882

[28] Jeon CY, Murray MB. Diabetes mellitus increases the risk of active tuberculosis: A systematic review of 13 observational studies. PLoS Med. C. Y. Jeon, Department of Epidemiology, Harvard School of Public Health, Boston, MA, United States; 2008;5: 1091–1101. Available: http://www.embase.com/search/results?subaction=viewrecord%7B&%7Dfrom=export%7B&%7Did=L352110389 10.1371/journal.pmed.0050152 PMC2459204 18630984

[29] Nanditha A, Ma RCW, Ramachandran A, Snehalatha C, Chan JCN, Chia KS, et al Diabetes in Asia and the Pacific: Implications for the Global Epidemic. Diabetes Care. 2016;39: 472–485. doi: 10.2337/dc15-1536 26908931 10.2337/dc15-1536

[30] Gupta S, Shenoy VP, Bairy I, Srinivasa H, Mukhopadhyay C. Diabetes mellitus and HIV as co-morbidities in tuberculosis patients of rural South India. J Infect Public Health. C. Mukhopadhyay, Department of Microbiology, Kasturba Medical College, Manipal University, Manipal 576104, Karnataka, India; 2011;4: 140–144. doi: 10.1016/j.jiph.2011.03.005 21843860 10.1016/j.jiph.2011.03.005

[31] Dooley KE, Chaisson RE. Tuberculosis and diabetes mellitus: convergence of two epidemics. Lancet Infect Dis. R.E. Chaisson, Division of Infectious Diseases, Center for Tuberculosis Research, Johns Hopkins University School of Medicine, Baltimore, MD, United States; 2009;9: 737–746. Available: http://www.embase.com/search/results?subaction=viewrecord%7B&%7Dfrom=export%7B&%7Did=L355760814 doi: 10.1016/S1473-3099(09)70282-8 19926034 10.1016/S1473-3099(09)70282-8 PMC2945809

[32] Ottmani S-E, Murray MB, Jeon CY, Baker MA, Kapur A, Lönnroth K, et al Consultation meeting on tuberculosis and diabetes mellitus: Meeting summary and recommendations. Int J Tuberc Lung Dis. A. D. Harries, Old Inn Cottage, Colden Common, Winchester SO21 1TQ, United Kingdom; 2010;14: 1513–1517. Available: http://www.embase.com/search/results?subaction=viewrecord%7B&%7Dfrom=export%7B&%7Did=L360026074 21180207

[33] Long R, Maycher B, Dhar A, Manfreda J, Hershfield E, Anthonisen N. Pulmonary tuberculosis treated with directly observed therapy: serial changes in lung structure and function. Chest. 1998;113: 933–943. 9554628 10.1378/chest.113.4.933

[34] Singla N, Singla R, Fernandes S, Behera D. Post treatment sequelae of multi-drug resistant tuberculosis patients. Indian J Tuberc. India; 2009;56: 206–212. 20469732

[35] Chiang CY. Associations between tobacco and tuberculosis. Int Lung Dis. 2007;11: 258–262. 17352089

